# Rectosigmoid Endometrioma Mimicking Rectal Hematoma: A Diagnostic Dilemma Managed Conservatively

**DOI:** 10.7759/cureus.100525

**Published:** 2025-12-31

**Authors:** Teng Yuen Ching Deborah, Ko-Ping Tiang, Mohd Syafferi Bin Masood

**Affiliations:** 1 Department of Surgery, Hospital Sungai Buloh, Sungai Buloh, MYS; 2 Department of Surgery, University Malaya Medical Centre, Kuala Lumpur, MYS

**Keywords:** endometriosis, gnrh analogue, hormonal therapy, rectal endometrioma, surgical case reports

## Abstract

Endometriosis is a benign gynecological condition characterized by the presence of functional endometrial tissue outside the uterine cavity. Although it most commonly affects pelvic organs, bowel involvement is uncommon, and rectal endometriosis is particularly rare. Its presentation may mimic colorectal malignancy, inflammatory bowel disease, or rectal hematoma, often resulting in diagnostic delay.

We report the case of a 38-year-old multiparous woman who presented with painless hematochezia, cyclical alteration of bowel habits, and lower abdominal cramping pain. Colonoscopy revealed a large intraluminal rectal lesion initially interpreted as a rectal hematoma. Computed tomography (CT) demonstrated a large intramural rectal mass with a concomitant left ovarian cystic lesion. Following multidisciplinary evaluation, a diagnosis of rectal endometrioma was favored. The patient was treated conservatively with gonadotropin-releasing hormone (GnRH) analogue therapy, resulting in the marked radiological regression of both rectal and ovarian lesions. Follow-up colonoscopy confirmed the near-complete resolution of the rectal lesion. The patient declined surgical intervention and remained clinically stable on maintenance hormonal therapy.

This case highlights the diagnostic challenge posed by rectal endometriosis and underscores the importance of considering endometriosis in reproductive-aged women presenting with atypical rectal symptoms. Magnetic resonance imaging (MRI) was used to confirm the diagnosis and to monitor treatment response, demonstrating the significant regression of the rectal lesion following hormonal therapy, and selected patients may be successfully managed with medical therapy alone.

## Introduction

Endometriosis is a benign gynecological disorder characterized by the presence of endometrial tissue outside the uterine cavity. Bowel endometriosis occurs in approximately 5%-12% of women with endometriosis, with the rectosigmoid colon accounting for up to 90% of bowel cases [1]. While pelvic organs are most commonly involved, rectal involvement is rare and can mimic colorectal malignancy, inflammatory bowel disease, or hematoma, frequently leading to misdiagnosis and delays in management. Common presentations of rectal endometriosis include cyclical alterations in bowel habits, dyschezia, dysmenorrhea, dyspareunia, bloating, nausea, and vomiting. We report a case of rectosigmoid endometrioma initially diagnosed as a rectal hematoma.

## Case presentation

**Figure 1:**
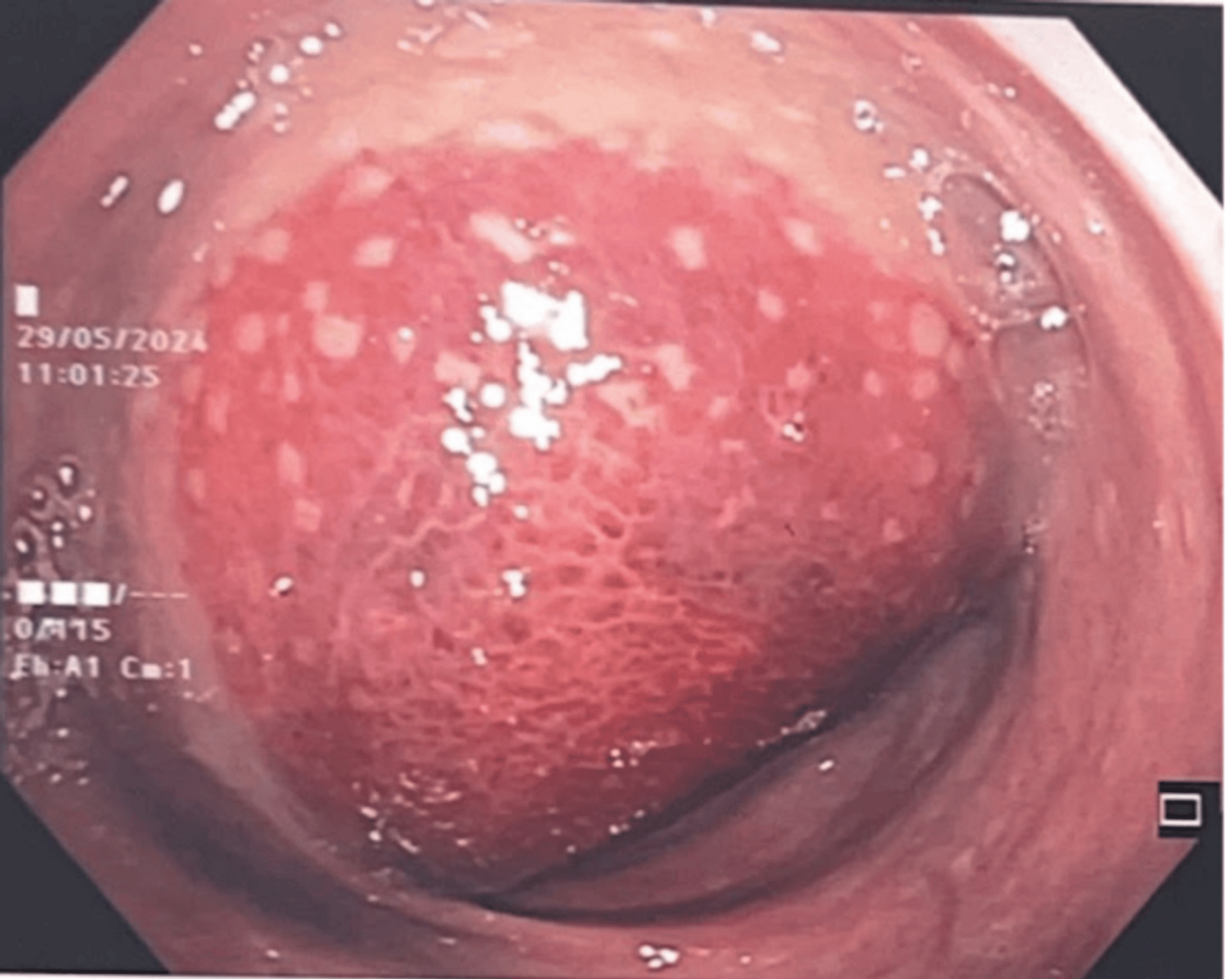
Colonoscopic image demonstrating a submucosal rectal lesion located 15-20 cm from the anal verge.

**Figure 2:**
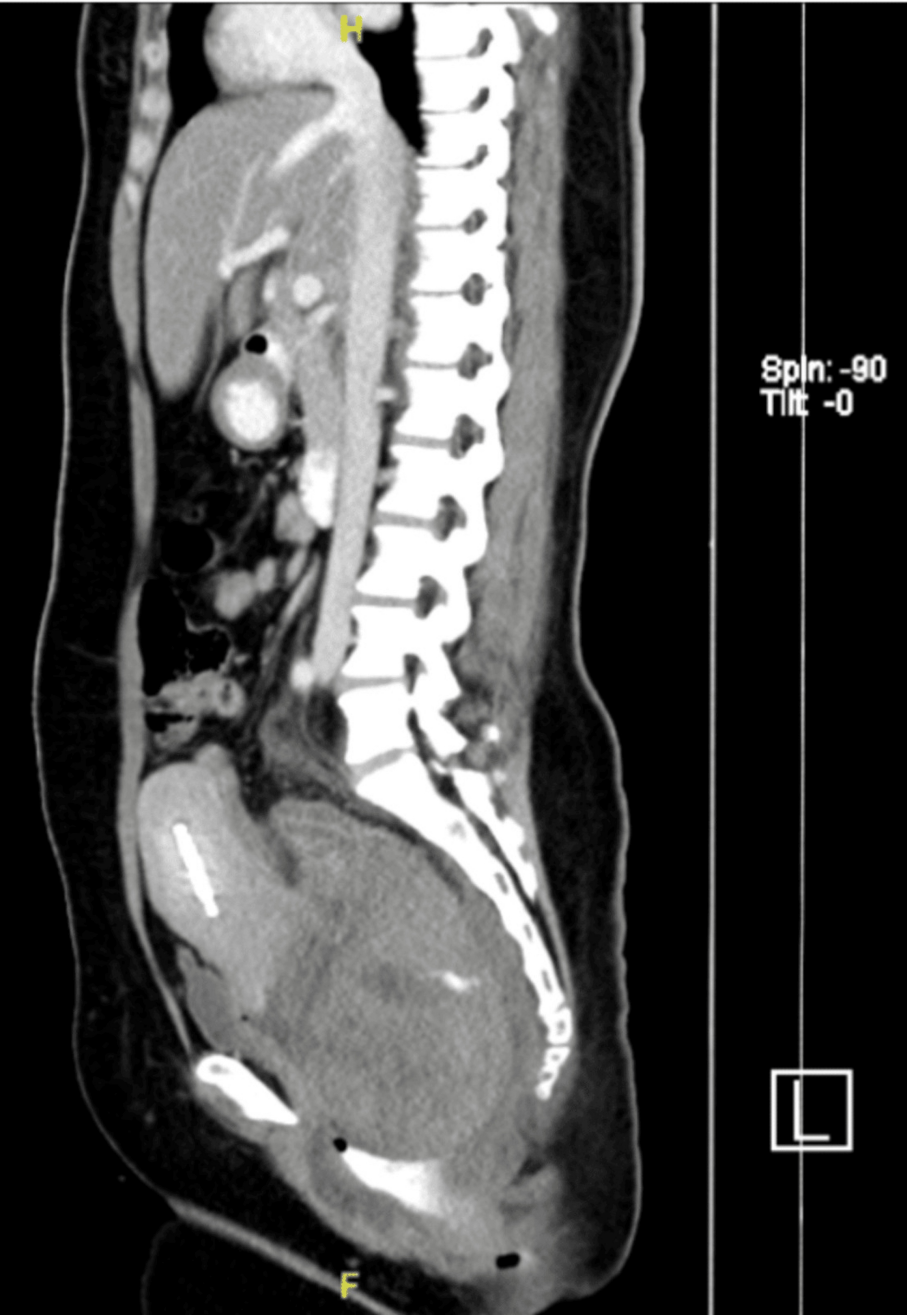
Sagittal contrast-enhanced computed tomography of the abdomen and pelvis showing a large intraluminal rectal mass initially interpreted as a rectal wall hematoma.

**Figure 3:**
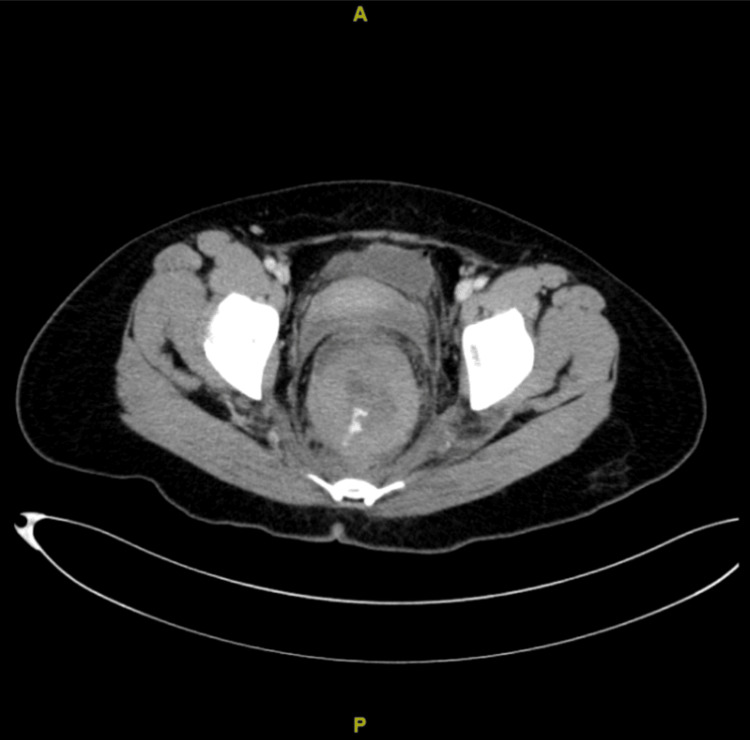
Axial contrast-enhanced computed tomography of the abdomen and pelvis demonstrating a large intraluminal rectal mass with evidence of active bleeding and an associated left ovarian cystic lesion.

A 38-year-old woman, para 5, was referred to our hospital for the evaluation of a rectal hematoma or adenocarcinoma. She reported regular menstrual cycles with associated dysmenorrhea of moderate severity, which had been managed conservatively with oral analgesics. There was no prior diagnosis of endometriosis. She had no history of previous gynecological surgery and had not received long-term hormonal therapy prior to presentation. There was no history of pelvic inflammatory disease or known gynecological malignancy. She presented with a single episode of painless hematochezia, cyclical alteration of bowel habits, and lower abdominal cramping pain. Colonoscopy revealed a large hematoma, and a repeat colonoscopy two weeks later noted a submucosal lesion of 15-20 cm from the anal verge (Figure 1) corresponding to the rectosigmoid region. Endoscopic biopsy was deferred not only due to the risk of bleeding but also because the lesion appeared submucosal with intact overlying mucosa, reducing the diagnostic yield of superficial biopsy. The computed tomography (CT) of the abdomen (Figures 2, 3) demonstrated an 8.2 × 8.1 × 12 cm intraluminal rectal mass, initially interpreted as a rectal wall hematoma, and a left ovarian cystic lesion measuring 5.5 × 5.7 × 5.5 cm.

**Figure 4:**
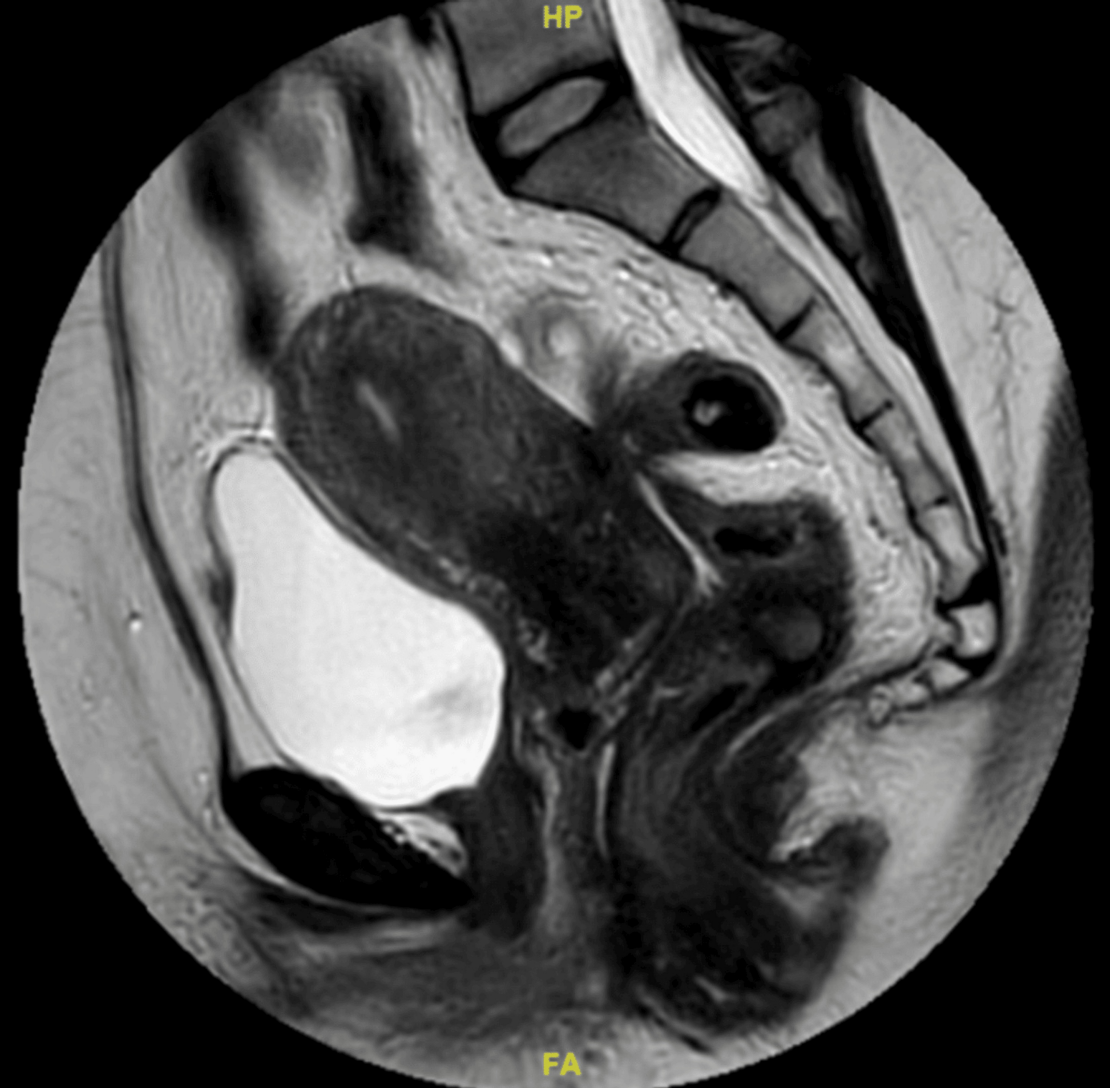
Sagittal magnetic resonance imaging of the pelvis following six doses of gonadotropin-releasing hormone analogue therapy, showing the marked regression of the rectal lesion.

**Figure 5:**
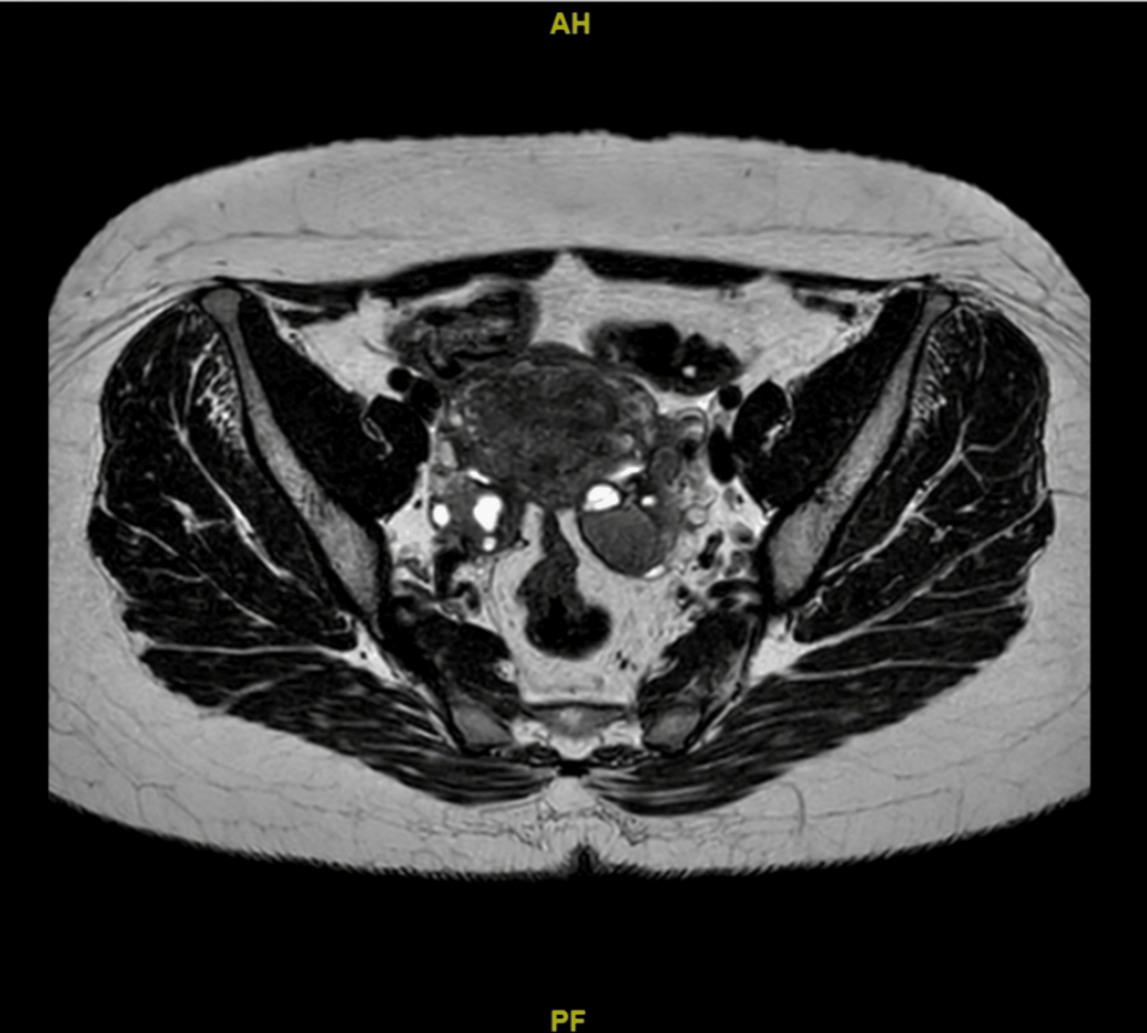
Axial magnetic resonance imaging of the pelvis after six doses of gonadotropin-releasing hormone analogue therapy, demonstrating the significant reduction of both the rectal endometrioma and the left ovarian endometrioma.

**Figure 6:**
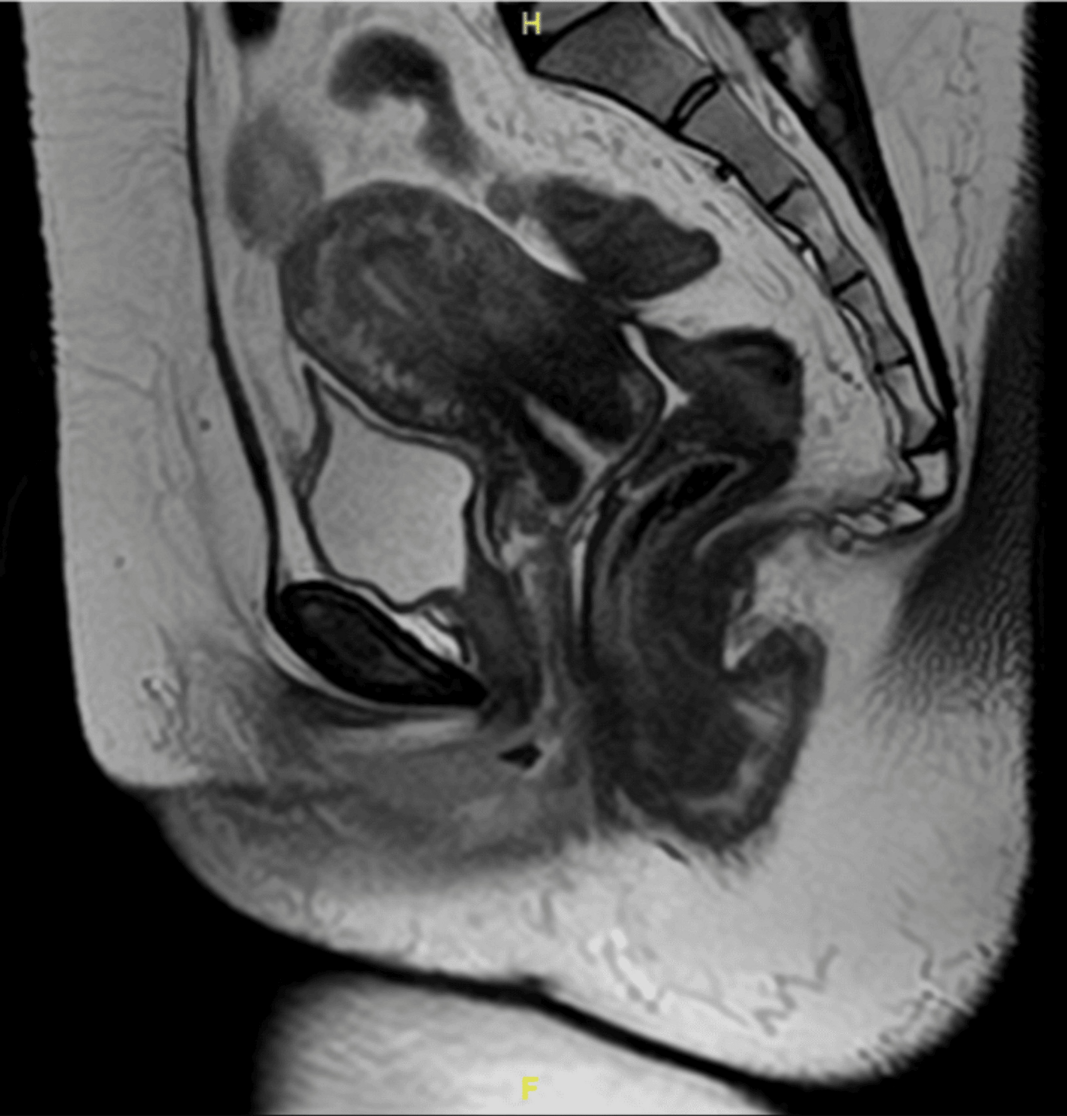
Sagittal magnetic resonance imaging of the pelvis performed six months after the completion of hormonal therapy.

**Figure 7:**
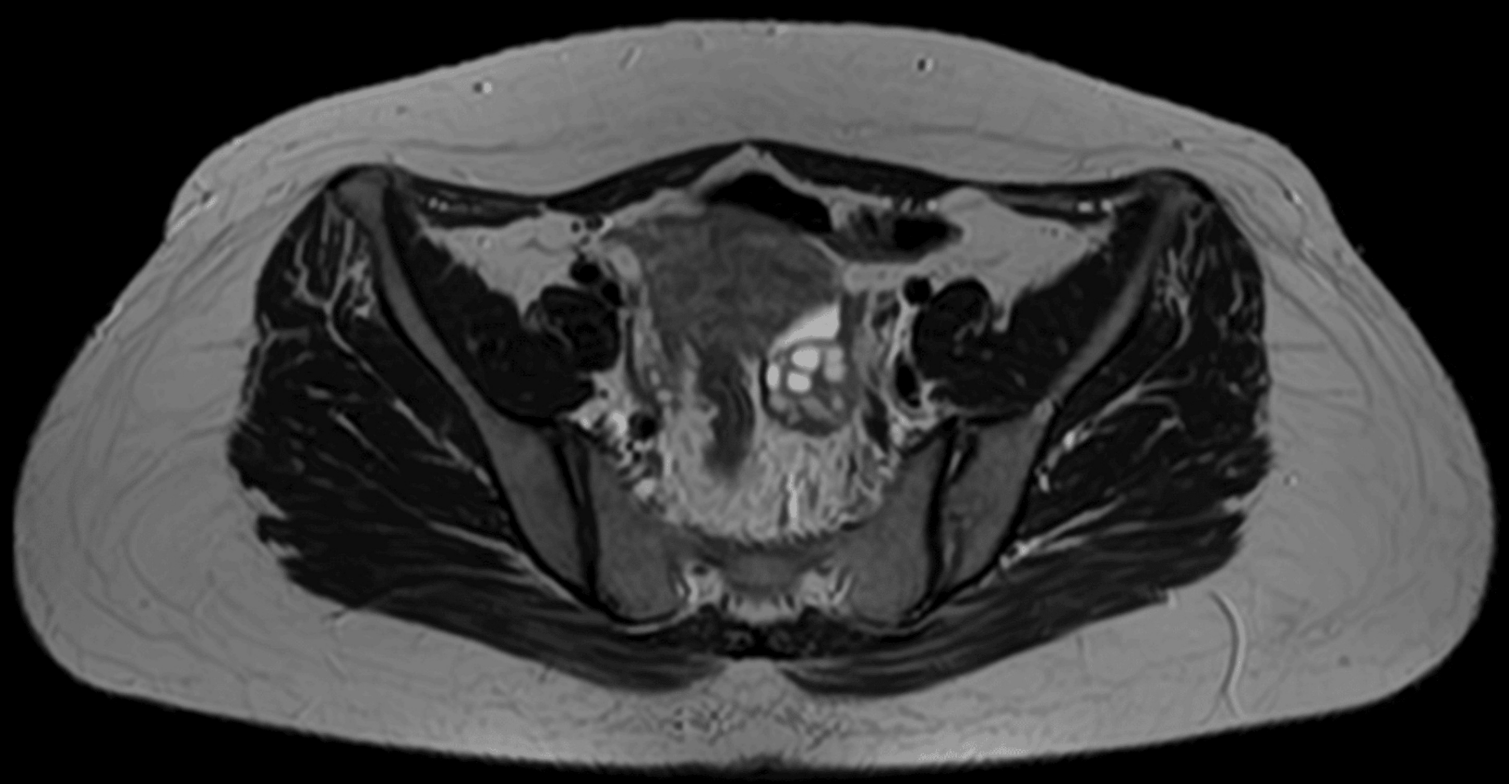
Axial magnetic resonance imaging of the pelvis six months after treatment, demonstrating a stable residual rectal lesion measuring 1.1 × 1.0 × 2.1 cm.

A multidisciplinary team (MDT) discussion concluded that the findings were most consistent with a rectal endometrioma. Conservative treatment with a gonadotropin-releasing hormone (GnRH) analogue administered monthly for a total duration of six months was initiated, followed by reassessment with magnetic resonance imaging (MRI) of the pelvis and colonoscopy. Add-back therapy was not initiated, as the planned duration of GnRH analogue therapy was limited to six months, and the patient was closely monitored for hypoestrogenic symptoms. Posttreatment MRI (Figures 4, 5) showed a dramatic reduction of both the rectal lesion (from 8.2 × 8.1 × 12 cm to 1.0 × 1.1 × 2.1 cm) and the left ovarian endometrioma (from 5.5 × 5.7 × 5.5 cm to 1.9 × 2.3 × 2.5 cm). A follow-up colonoscopy confirmed the resolution of the lesion, showing only mild rectal fold thickening. Although a total abdominal hysterectomy and bilateral salpingo-oophorectomy (TAHBSO) was planned, the patient, satisfied with symptomatic improvement, declined surgery and continued treatment with dienogest (Visanne). Repeat MRI six months later demonstrated a stable residual lesion measuring 1.1 × 1.0 × 2.1 cm (Figures 6, 7). Following the completion of GnRH analogue therapy, dienogest was continued as maintenance treatment to suppress residual disease, prevent recurrence, and maintain symptomatic control. This approach was chosen to avoid prolonged hypoestrogenic effects associated with extended GnRH use while providing effective long-term hormonal suppression. The patient tolerated dienogest well and remained clinically stable, with no recurrence of rectal bleeding or worsening gastrointestinal symptoms up to three months of follow-up.

The patient was satisfied with the symptom improvement achieved through medical management and expressed a preference for avoiding surgical intervention. She valued the multidisciplinary approach and close follow-up that supported her treatment decisions.

## Discussion

This case illustrates the diagnostic complexity of rectosigmoid endometrioma presenting with atypical rectal bleeding and imaging features suggestive of a rectal hematoma. Unlike typical presentations characterized by dyschezia or obstructive symptoms, this patient presented with painless hematochezia, contributing to initial diagnostic uncertainty. The differential diagnosis of a large intramural rectosigmoid mass includes colorectal carcinoma, rectal wall hematoma, gastrointestinal stromal tumor (GIST), inflammatory bowel disease, endometriosis, and, less commonly, vascular malformations. In this case, the absence of systemic symptoms, intact mucosa on colonoscopy, and cyclical gastrointestinal symptoms and the presence of a concurrent ovarian endometrioma favored a benign gynecological etiology over malignant or inflammatory causes. Similar cases of bowel endometriosis masquerading as colorectal malignancy or hematoma have been reported, most often requiring surgical exploration for diagnosis. In contrast, this case demonstrates that selected patients with characteristic imaging findings and stable clinical features may be successfully managed conservatively, avoiding unnecessary surgery [2,3]. Rectosigmoid endometrioma is a form of deep infiltrating endometriosis (DIE), characterized by the ectopic implantation and invasion of endometrial glands and stroma into the bowel wall, often extending through the muscularis propria while sparing the mucosa. Several mechanisms have been proposed for its pathogenesis. Retrograde menstruation may allow endometrial tissue to reach the serosal surface of the rectosigmoid colon. Coelomic metaplasia and lymphovascular or neural dissemination have also been suggested as contributing factors, particularly in cases without pelvic involvement. The chronic cyclical hormonal stimulation of ectopic endometrial tissue leads to repeated hemorrhage, fibrosis, and smooth muscle hypertrophy, which collectively produce the submucosal mass effect observed on imaging. These pathophysiological processes explain both the typical submucosal location and the often-normal overlying mucosa on colonoscopy, contributing to diagnostic difficulty [4].

Rectal endometriosis can mimic colorectal malignancy, gastrointestinal stromal tumors (GIST), or inflammatory disease such as Crohn's disease or vascular malformations such as rectal varices. Typical symptoms include constipation, dyschezia, tenesmus, and cyclical rectal bleeding. As in this case, unusual presentations such as painless rectal bleeding without overt pain may mislead clinicians [5].

The histological confirmation of bowel endometriosis is often challenging, as the disease typically involves the muscularis propria while sparing the mucosa, rendering endoscopic biopsy frequently nondiagnostic. In selected cases with characteristic imaging findings and typical clinical features, a multidisciplinary, non-invasive diagnostic approach may be appropriate. Radiological imaging is crucial in evaluation. While CT and CT angiography are helpful to exclude bleeding or vascular malformations, they are nonspecific for endometriotic lesions [6]. Magnetic resonance imaging is the preferred non-invasive modality for the evaluation of suspected deep infiltrating endometriosis, particularly for assessing lesion extent and treatment response, although definitive diagnosis may still require histological confirmation in selected cases [7,8]. Characteristic MRI features, such as T1-weighted hyperintensity and T2 shading, reflect chronic hemorrhagic content within endometriotic foci and help distinguish them from neoplastic or inflammatory lesions [9]. In this patient, MRI findings, together with cyclical gastrointestinal symptoms and a concomitant left ovarian endometrioma, strongly supported the diagnosis of rectal endometriosis.

Management depends on symptom severity, lesion extent, and patient preference. Consensus guidelines recommend hormonal suppression as first-line therapy for mild or regressing disease, using agents such as GnRH analogues, progestins (e.g., dienogest), or combined oral contraceptives [7-9]. Surgery is reserved for cases with persistent symptoms, progressive lesions, or bowel obstruction, with options including lesion shaving, discoid excision, or segmental resection [10]. In this case, marked regression following medical therapy supported a conservative, nonsurgical approach. This aligns with growing evidence favoring individualized medical management and highlights the importance of multidisciplinary collaboration between gynecology, colorectal surgery, and radiology.

## Conclusions

Rectosigmoid endometrioma is a rare and challenging diagnosis due to its varied presentation and resemblance to colorectal malignancy. This case underscores the importance of considering endometriosis in women of reproductive age presenting with cyclical rectal or gastrointestinal symptoms. MRI remains the gold standard for diagnosis and follow-up. Hormonal therapy can achieve significant regression in selected patients, while surgery should be reserved for refractory or obstructive cases. A multidisciplinary approach is crucial for accurate diagnosis and optimal outcomes.
